# Novel compound heterozygous *POLR3A* variation: expanding 4H syndrome with progressive keratoconus

**DOI:** 10.1210/jcemcr/luag333

**Published:** 2026-11-06

**Authors:** Enver Simsek, Tulay Simsek, Oguz Cilingir, Eray Atalay

**Affiliations:** Division of Pediatric Endocrinology, Department of Pediatrics, Eskişehir Osmangazi University School of Medicine, Eskişehir 26040, Türkiye; Department of Ophthalmology, Eskişehir Osmangazi University School of Medicine, Eskişehir 26040, Türkiye; Department of Medical Genetics, Eskişehir Osmangazi University School of Medicine, Eskişehir 26040, Türkiye; Department of Ophthalmology, Eskişehir Osmangazi University School of Medicine, Eskişehir 26040, Türkiye

**Keywords:** *POLR3A*, 4H syndrome, keratoconus, hypogonadotropic hypogonadism, penetrating keratoplasty, collagen cross-linking

## Abstract

*POLR3A*-related leukodystrophy is a multisystem disorder classically defined by hypomyelination, hypodontia, and hypogonadotropic hypogonadism (4H syndrome). While neurological and endocrine features are well-defined, the ocular phenotypic spectrum remains incomplete. We present a 15.9-year-old female with a classic 4H phenotype and compound heterozygosity for 2 novel variants (NM_007055.4:c.2102G > T [p.(Gly701Val)] and NM_007055.4:c.2989C > T [p.(Pro997Ser)]). The patient exhibited advanced, rapidly progressive bilateral keratoconus, with a maximum keratometry (*K*-max) reaching an extreme 75.9 diopter (D). *K*-max of 75.9 D (approximately 40-47 D in children and adolescents) represents extreme corneal ectasia. Longitudinal follow-up documented progressive ectasia, treated with penetrating keratoplasty in the left eye and corneal collagen cross-linking in the right eye. On the postoperative Pentacam examination at age 22, right-eye *K*-max was 70.7 D (66.7 D at age 10), and the thinnest location was 304 µm; left-eye values were unreliable owing to automated quality alerts. This case highlights progressive keratoconus as a potential clinical component of *POLR3A*-related disorders. We propose that ribonucleic acid polymerase III is essential for corneal proteostasis. Our findings position keratoconus as a potential early clinical sentinel for 4H syndrome, necessitating mandatory corneal topography in suspected cases to ensure timely, vision-saving intervention.

## Introduction

*POLR3*-related leukodystrophy is a rare autosomal recessive neurodegenerative disorder that has undergone significant clinical and molecular refinement over the past 2 decades. The condition was first clinically delineated in 2005 as a distinct hypomyelinating entity [1]. The molecular breakthrough followed in 2011, when biallelic variations in *POLR3A* and *POLR3B*, encoding the catalytic subunits of ribonucleic acid (RNA) polymerase III (Pol III), were identified as the underlying cause [2, 3]. This multisystem disorder, widely recognized as 4H (hypomyelination, hypodontia, and hypogonadotropic hypogonadism) syndrome, is classically defined by a hallmark clinical triad: hypomyelination associated with progressive cerebellar ataxia, hypodontia with delayed or abnormal dentition, and hypogonadotropic hypogonadism (HHG) [4, 5].

While the neurological decline and endocrine failures are well characterized, the phenotypic boundaries of *POLR3*-related disorders continue to expand. Historically, ocular involvement has been considered a secondary feature, primarily limited to high myopia, or more rarely, cataracts and optic atrophy [5]. However, because Pol III is essential for transcribing small, noncoding RNAs (nc-RNAs) required for fundamental cellular processes, its dysfunction is now understood to impair various ectodermal and mesenchymal tissues [6, 7]. Recent evidence suggests that the spectrum may include broader structural and connective tissue abnormalities, challenging the traditional limits of the 4H phenotype [8].

In this report, we present a 15.9-year-old female with a novel compound heterozygous *POLR3A* variant and a classic 4H presentation. We document the presence of advanced, rapidly progressive bilateral keratoconus, reaching an extreme maximum keratometry (*K*-max) of 75.9 diopters (D), as a potential clinical feature of *POLR3A*-related disease. By integrating longitudinal corneal topography with genetic findings, we further explore the potential relationship between *POLR3* dysfunction and corneal structural integrity [9]. We further report postoperative Pentacam findings after penetrating keratoplasty (PK) in the left eye and corneal collagen cross-linking (CXL) in the right eye [10, 11].

## Case presentation

A 15.9-year-old female was referred for lack of pubertal progression and progressive gait instability. Her history included visual impairment from age 7 and balance disorders from age 9. Despite early adrenarche, she presented with primary amenorrhea and Tanner stage 1 breast development. Family history was notable for first-cousin consanguinity (Fig. 1).

**Figure 1:**
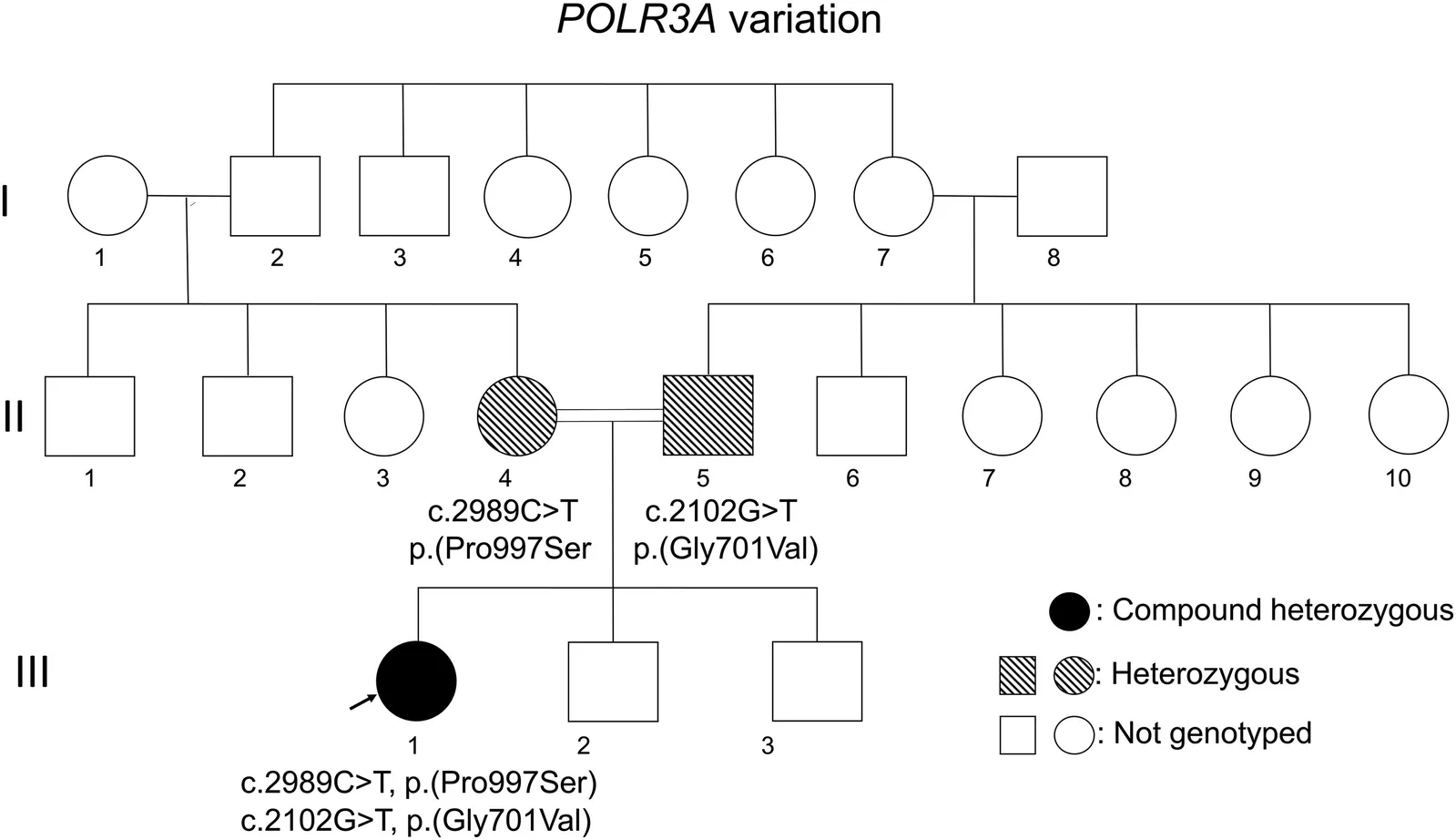
Pedigree of the family with *POLR3A* variants. The pedigree demonstrates autosomal recessive inheritance in a consanguineous family (first-cousin marriage indicated by double horizontal lines). The proband (III-1, arrow) is compound heterozygous for c.2102G>T (p.Gly701Val) and c.2989C>T (p.Pro997Ser). The mother (II-4) carries p.Pro997Ser and the father (II-5) carries p.Gly701Val. Filled circle = affected individual (compound heterozygous); hatched symbols = heterozygous carriers; open symbols = unaffected individuals; arrow = proband; double horizontal line = consanguineous union; Roman numerals = generations; Arabic numerals = individual numbers within generations.

Physical examination revealed a height of 153 cm (−1.6 standard deviation score [SDS]), a weight of 44 kg (−2.1 SDS), and a body mass index of 18.8 kg/m^2^. Dental examination confirmed hypodontia (absent maxillary premolars).

## Diagnostic assessment

Laboratory investigations established profound HHG. Basal follicle-stimulating hormone and luteinizing hormone levels were both <0.3 mIU/mL (<0.3 U/L; reference ranges, 0.4-8.6 mIU/mL [0.4-8.6 U/L] for follicle-stimulating hormone and 0.9-13.3 mIU/mL [0.9-13.3 U/L] for luteinizing hormone), whereas estradiol was <5 pg/mL (<18.4 pmol/L; reference range, 15-350 pg/mL [55-1285 pmol/L]).

Whole-exome sequencing was performed because of a syndromic multisystem phenotype suggestive of a genetic leukodystrophy spectrum disorder. Analysis identified compound heterozygous variants in *POLR3A* (NM_007055.4:c.2102G > T [p.(Gly701Val)] and NM_007055.4:c.2989C > T [p.(Pro997Ser)]), which were confirmed by segregation analysis and were consistent with autosomal recessive inheritance (Fig. 1).

Variant interpretation was performed in accordance with American College of Medical Genetics and Genomics/Association for Molecular Pathology guidelines. In silico prediction tools (including SIFT, PolyPhen-2, and MutationTaster) provided supportive evidence of a deleterious effect (PP3); however, these computational data were not used as standalone evidence for pathogenicity classification. Variant interpretation was based on an integrated assessment, including phenotypic concordance with 4H syndrome, compound heterozygosity in a recessive disorder, and population database filtering. Accordingly, we classified the variants as likely pathogenic within the American College of Medical Genetics and Genomics/Association for Molecular Pathology framework.

Cranial magnetic resonance imaging, performed at age 13 years on a 3-Tesla scanner (Fig. 2A–2E), demonstrated characteristic POLR3-related leukodystrophy features. T_2_- and T_1_-weighted images (Fig. 2A and 2B) revealed diffuse deep cerebral white matter hyperintensity, nearly isointense to the cortex on T_1_, characteristic of hypomyelination. Selective sparing of the ventrolateral thalamic (Fig. 2C) and dentate nuclei (Fig. 2D) provided a specific radiological signature of *POLR3A* variants. Atrophy of the brainstem, middle cerebellar peduncles, and cerebellar vermis (Fig. 2D and 2E) was consistent with the patient's ataxia.

**Figure 2:**
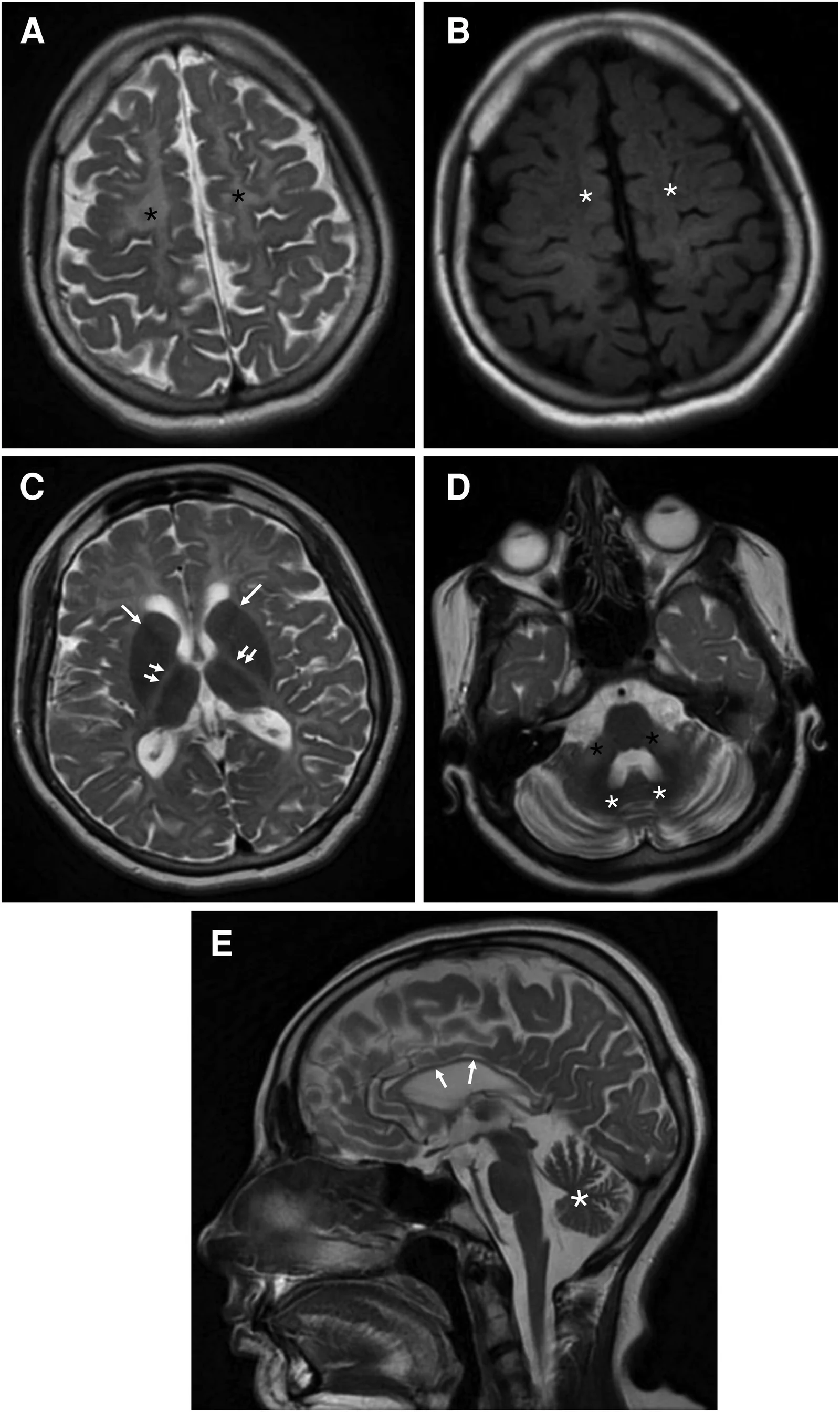
Cranial magnetic resonance imaging characteristics of *POLR3A*-related leukodystrophy. A comprehensive spectrum of hypomyelination and infratentorial atrophy is demonstrated across multiple planes. (A, B) Hypomyelination pattern: Axial T_2_-weighted (A) and T_1_-weighted (B) images reveal diffuse signal abnormality of the deep cerebral white matter. On the T_2_-weighted sequence (A), diffuse hyperintensity is observed (black asterisks). Critically, on the T_1_-weighted sequence (B), the white matter is nearly isointense to the cortex (white asterisks), representing the characteristic hallmark of hypomyelination. (C) Selective sparing: The axial T_2_-weighted image demonstrates characteristic preservation of signal intensity in the ventrolateral thalamic nuclei (double arrows) and in the internal capsule (single arrows)—the anterior limb of which could not be reliably delineated owing to volume expansion of the corpus striatum. This selective sparing of these structures, contrasting with the surrounding hypomyelination, represents a specific and characteristic radiological signature of pathogenic *POLR3A* variants. (D, E) Atrophy and neurodegeneration: Axial T_2_-weighted (D) and sagittal T_2_-weighted (E) images illustrate significant volume loss throughout the infratentorial and midline structures. (D) Notable findings include significant atrophy of the brainstem and middle cerebellar peduncles (black asterisks), with relative preservation of the dentate nuclei (white asterisks). (E) Sagittal view reveals marked thinning of the corpus callosum (white arrows) and prominent cerebellar vermian atrophy (white asterisk), correlating with the patient's progressive ataxia and motor deterioration.

## Treatment

For HHG, cyclic hormone replacement (transdermal estradiol and oral progesterone) was initiated at age 15.9, leading to withdrawal bleeding at the age of 16.5 years. The patient never had spontaneous menses, and the absence of menstrual bleeding is to be expected in the absence of hormone replacement therapy.

## Outcome and follow-up

Ophthalmological follow-up revealed exceptionally aggressive keratoconus. At age 7, *K*-max was 55.0 D in the right eye (OD) and 61.6 D in the left eye (OS; Fig. 3, Table 1). By age 10, OS *K*-max reached an extreme of 75.9 D (Fig. 4). The left-eye examination at age 10 carried automated quality alerts (“Data G” for the front surface and “Model!” for the back surface); the *K*-max of 75.9 D should therefore be interpreted with caution. It is, however, accompanied by a steep K2 of 68.6 D, a mean keratometry of 63.8 D, a minimum front radius of 4.45 mm, and a thinnest location of 318 µm on the same examination, and it follows the progression seen in the right eye. *K*-max of 75.9 D (normal range in children and adolescents approximately 40-47 D) indicated severe corneal ectasia. At age 22, due to imminent perforation risk, the patient underwent PK in the left eye and standalone corneal collagen CXL in the right eye (*K*-max 66.7 D at age 10) to halt ectasia. The postoperative Pentacam examination (12 September 2025) showed, in the right eye, a *K*-max of 70.7 D, a thinnest location of 304 µm, and front astigmatism of 9.5 D (Table 1, Fig. 5). In the grafted left eye, automated quality alerts (Blinking/Model!) were present, and the device did not identify a thinnest location; the displayed values were considered unreliable (Table 1, Fig. 5). The patient, now 23, continues multidisciplinary follow-up.

**Figure 3:**
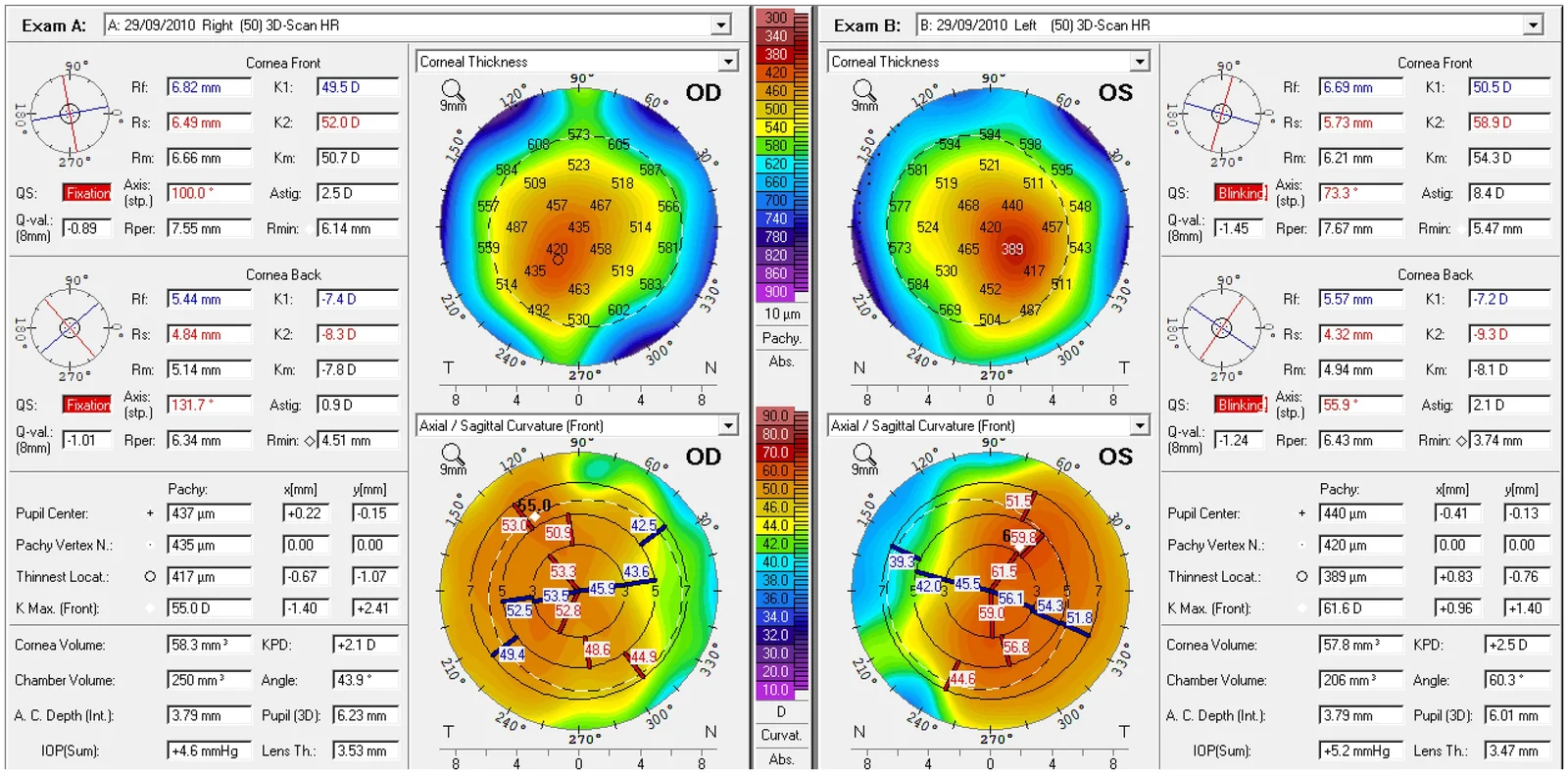
Initial corneal topography (Pentacam HR) at age 7. Baseline assessment reveals bilateral advanced keratoconus in early childhood. Color-coded curvature analysis: Axial curvature maps demonstrate pathological steepening represented by warm colors (orange/red). In the left eye (OS), the maximum keratometry (*K*-max) is pathologically high at 61.6 D, while the right eye (OD) shows 55.0 D. Pachymetric thinning: Thickness maps indicate significant structural weakness. The thinnest corneal locations are 389 µm in the left eye (OS) and 417 µm in the right eye (OD), both significantly below the physiological range of 530-560 µm. Global indices: The topography shows characteristic inferior-nasal steepening and central stromal thinning, confirming early-onset, aggressive corneal ectasia.

**Figure 4:**
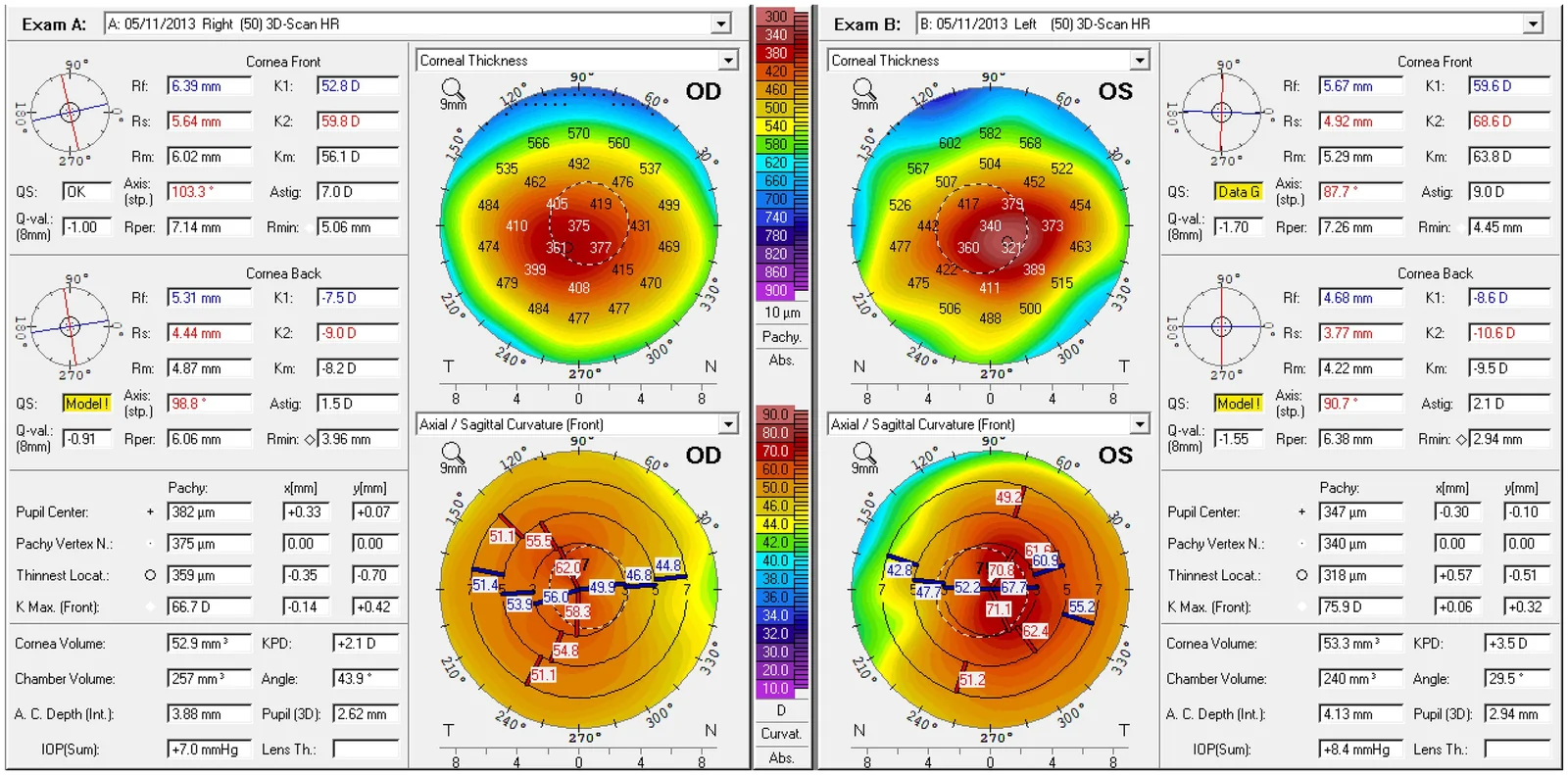
Longitudinal evaluation of corneal surface morphometry. Disease progression and structural collapse at age 10. Follow-up imaging demonstrates an exceptionally aggressive clinical course over a 3-year interval. Extreme topographic progression: Axial curvature maps show that central “heat zones” have intensified into dark red, indicating a rapid escalation of K-max to 75.9 D in the left eye (OS) and 66.7 D in the right eye (OD). Automated software alerts: Yellow quality-status (QS) alerts are displayed: “Data G” (front surface) and “Model!” (back surface) in the left eye, and “Model!” (back surface) in the right eye. These are automated data-quality flags (incomplete data acquisition and model-based reconstruction in a highly irregular cornea); they are not measures of disease severity, and the left-eye values, including *K*-max 75.9 D, should be interpreted with caution. Critical pachymetric thinning: The structural integrity has further deteriorated, with the thinnest corneal location reaching a dangerously low level of 318 µm in the left eye (OS) and 359 µm in the right eye (OD). These findings indicate a state at high risk of corneal perforation.

**Figure 5:**
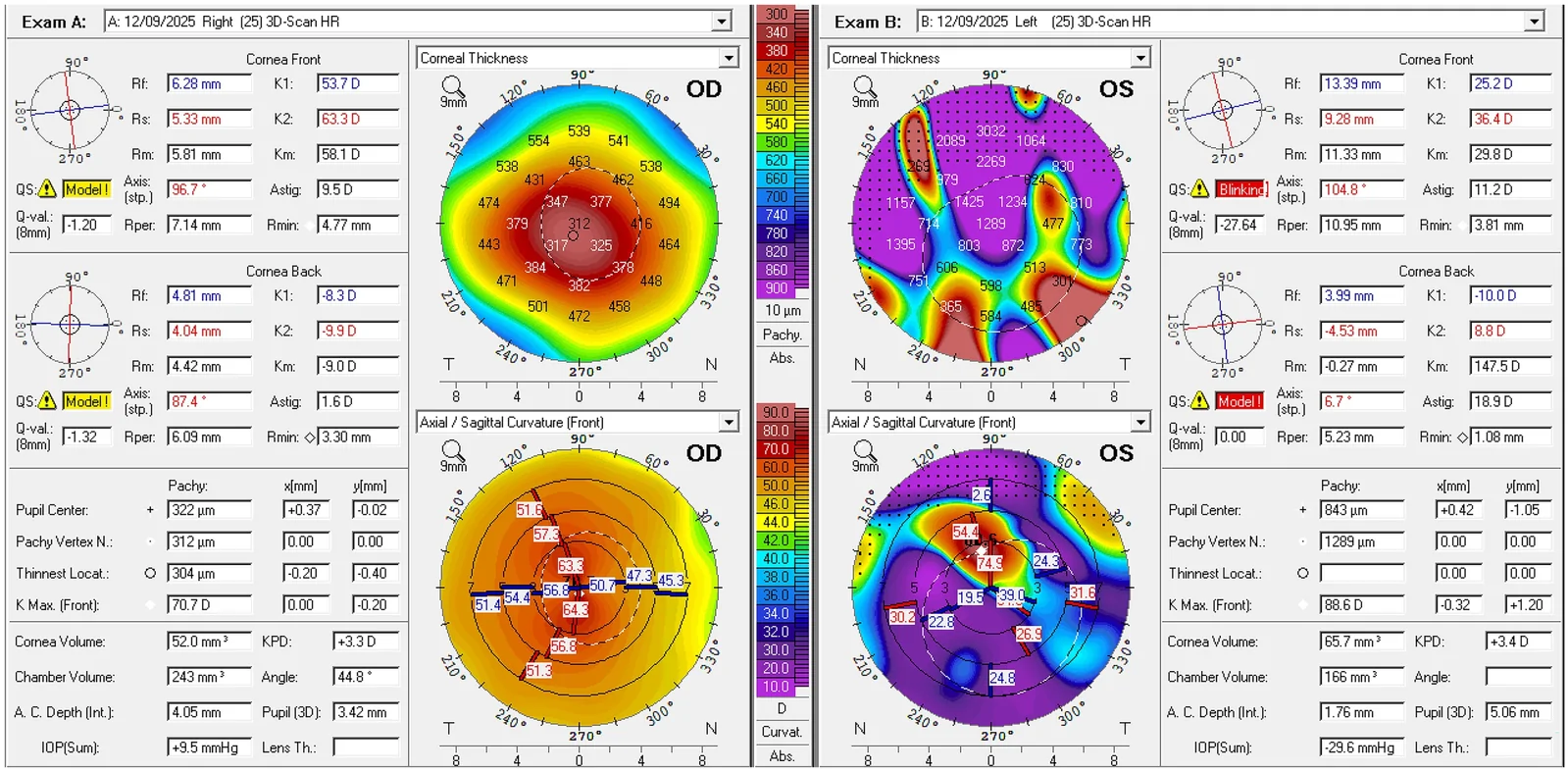
Pentacam HR output at age 22 (12 September 2025), after penetrating keratoplasty in the left eye (OS) and collagen cross-linking (CXL) in the right eye (OD). Left eye (OS): Automated quality alerts (Blinking, Model!) are present, and the device did not identify a thinnest location; the displayed values, including a *K*-max of 88.6 D, are not considered reliable in the grafted cornea. Right eye (OD): *K*-max 70.7 D, thinnest location 304 µm, front astigmatism 9.5 D, and corneal volume 52.0 mm^3^. As in Fig. 4, a “Model!” alert is present, consistent with the severity of the underlying ectasia.

**Table 1:** Longitudinal Pentacam measurements before and after penetrating keratoplasty (OS) and corneal collagen CXL (OD)

Parameter	Age 7		Age 10		Age 22*^a^*		Reference range
	(OD)	(OS)	(OD)	(OS)	(OD)	(OS)	
*K*-max (front)	55.0 D	61.6 D	66.7 D	75.9 D	70.7 D	88.6 D*^b^*	40.0-47.0 D
Thinnest location	417 µm	389 µm	359 µm	318 µm	304 µm	—	530-560 µm
Astigmatism	2.5 D	8.4 D	7.0 D	9.0 D	9.5 D	11.2 D*^b^*	<1.0 D
Corneal volume	58.3 mm^3^	57.8 mm^3^	52.9 mm^3^	53.3 mm^3^	52.0 mm^3^	65.7 mm^3^*^b^*	58.0-60.0 mm^3^

Astigmatism refers to front-surface corneal astigmatism. Automated QS alerts were displayed on the Pentacam printouts for the left eye at age 10 (Data G/Model!) and at age 22 (Blinking/Model!).

Abbreviations: µm, micrometers; D, diopters; *K*-max, maximum keratometry (front); OD, oculus dexter; OS, oculus sinister; QS, Pentacam quality status.

^*a*^Unreliable: the left-eye (grafted cornea) examination at age 22 was acquired with QS alerts (Blinking/Model!); these values are shown for completeness only and should not be interpreted. —, not determined (the device did not identify a thinnest location).

^*b*^Postoperative 6-month follow-up (12 September 2025): corneal collagen CXL (OD) and penetrating keratoplasty (OS).

## Discussion

The *POLR3A* gene encodes the largest catalytic subunit of RNA Pol III, essential for transcribing small nc-RNAs, including tRNAs and 5S rRNA [12, 13]. The hypomyelinating phenotype in our patient stems from a defect in cellular “proteostasis.” A central paradox is why mutations in a ubiquitously expressed enzyme lead to tissue-specific phenotypes primarily affecting myelin and ectodermal derivatives [2, 5]. Oligodendrocytes exhibit unique metabolic vulnerability; during peak myelination, they must synthesize vast quantities of proteins like myelin basic protein. The novel compound heterozygous variants identified (p.(Gly701Val) and p.(Pro997Ser)) impair Pol III complex assembly or recruitment, reducing the tRNA pool [14]. This scarcity acts as a rate-limiting step, causing maturation arrest in oligodendrocytes and subsequent myelin failure [15].

Chronic deficiency in Pol III–regulated transcripts likely leads to cumulative cellular stress, triggering secondary neurodegeneration and global volume loss observed in our neuroimaging [5]. This proteostatic failure provides a mechanistic bridge to structural abnormalities in other ectodermal tissues, such as the cornea [9].

Neuroimaging (Fig. 2) distinguishes hypomyelination from demyelination. In our case, T_1_-weighted images show supratentorial white matter nearly isointense to gray matter, a pathognomonic hallmark [1, 4]. The “radiological signature” of *POLR3A* involves selective sparing of the ventrolateral thalamic and dentate nuclei, differentiating it from disorders like Pelizaeus–Merzbacher syndrome [16]. Wiedemann–Rautenstrauch syndrome has also been associated with biallelic variants in *POLR3A* and related genes, expanding the phenotypic spectrum of POLR3-related disorders [17-19]. Reported cases have demonstrated multisystem involvement, including growth failure, progeroid features, and in some instances, ocular abnormalities such as central corneal thinning [17, 19]. These corneal structural changes may show partial morphological overlap with ectatic disorders, such as keratoconus; however, whether this represents a shared pathogenic mechanism or phenotypic convergence remains unclear [19]. Collectively, these observations further support the broad and variable ectodermal involvement in POLR3-related disease, while emphasizing that corneal ectasia has not been systematically established as a core feature of this spectrum [17-19]. Significant cerebellar atrophy and thinning of the corpus callosum are consistent with the patient's clinical deterioration from age 9 [5, 20].

Hypogonadotropic hypogonadism is a cardinal feature of 4H syndrome associated with *POLR3* variants, evidenced by a flat gonadotropin-releasing hormone (GnRH) response [21]. Efficient Pol III transcription is essential for nc-RNAs regulating GnRH synthesis and release. A quantitative tRNA deficiency likely prevents specialized neurons from sustaining the proteostatic environment required for puberty [14, 22]. In POLR3-related disease, the selective involvement of the gonadotropic axis is thought to reflect the developmental vulnerability of GnRH neuronal networks to RNA Pol III dysfunction. In contrast, other anterior pituitary hormone axes are typically preserved, suggesting a preferential effect on hypothalamic reproductive regulatory pathways rather than diffuse pituitary impairment. Furthermore, the growth failure (height SDS −1.6) is primarily attributable to the absence of the pubertal growth spurt and lost synergism between sex steroids and the growth hormone axis [8, 23].

The most distinctive feature of this case is advanced, rapidly progressive bilateral keratoconus, which was identified before the overt neurological manifestations. Ocular involvement in *POLR3*-related disorders has traditionally been limited to high myopia or cataracts [5]. However, emerging evidence suggests that the ocular phenotype may be broader than previously appreciated. The cornea, as a derivative of both surface ectoderm and neural crest–derived tissues, may represent a susceptible but under-recognized target in disorders of RNA Pol III dysfunction. The structural integrity of the corneal stroma depends on a tightly regulated extracellular matrix composed of collagen fibrils and proteoglycans, which is essential for maintaining biomechanical stability [9].

In our patient, the severity of keratoconus, with a *K*-max of 75.9 D and marked stromal thinning (318 µm), reflects profound corneal biomechanical failure. We hypothesize that the biallelic *POLR3A* variants identified in this patient (p.(Gly701Val) and p.(Pro997Ser)) may contribute to impaired transcription of small noncoding RNAs involved in cellular homeostasis and extracellular matrix maintenance. A relative imbalance in tRNA availability may result in a translational stress response in keratocytes, potentially affecting their ability to maintain corneal stromal integrity [14, 24].

The clinical course further illustrates differential tissue susceptibility within ectoderm-derived structures. In the left eye, PK was performed because of imminent perforation risk. In the right eye, CXL was performed; the postoperative *K*-max was 70.7 D, and the effect of CXL on disease progression cannot be established from a single postoperative Pentacam examination [10, 11]. Taken together, these findings suggest that corneal ectasia may represent a potential, but as yet unconfirmed, component of the expanding ectodermal phenotype associated with *POLR3A/B*-related disorders, which classically include hypomyelination, hypodontia, and HHG [25, 26]. While this observation raises the possibility of ocular involvement beyond previously recognized manifestations, the limited number of reported cases precludes definitive conclusions regarding its specificity or diagnostic utility [27]. Accordingly, keratoconus should currently be considered an exploratory phenotypic association rather than a screening indicative or diagnostic feature of 4H syndrome [28].

The novel variants (c.2102G > T and c.2989C > T) expand the 4H syndrome genotypic landscape. Complete loss-of-function *POLR3* variants are lethal; survival requires “leaky” compound heterozygosity [28, 29]. This molecular vulnerability may help explain why tissues with high protein-synthesis demands, such as myelinating oligodendrocytes, are most severely affected [15]; whether collagen-producing keratocytes are similarly susceptible remains speculative.

In conclusion, this case provides detailed documentation of advanced, rapidly progressive keratoconus in a patient with *POLR3A*-related leukodystrophy. Our findings expand the understanding of ocular involvement in POLR3-related disorders, as ocular manifestations have been reported in some cases but may be absent in others. The response to CXL in this setting could not be established from a single postoperative measurement and warrants further study. We propose integrating corneal topography into the multidisciplinary follow-up of all patients with suspected *POLR3*-related disorders to screen for ocular manifestations and, if indicated, ensure early intervention to reduce the risk of visual impairment.

## Learning points

- In a patient with classic 4H syndrome, we documented severe, early-onset bilateral keratoconus that progressed rapidly over 3 years (*K*-max 55.0/61.6 D at age 7 to 66.7/75.9 D at age 10 in the right/left eye). Whether keratoconus is a true component of *POLR3A*-related disease remains unconfirmed, and a single case cannot establish this association.
- Ocular involvement in POLR3-related disorders has traditionally been limited to myopia, cataract, and optic atrophy. Impaired RNA Pol III function may plausibly affect corneal stromal homeostasis, but this mechanism is speculative and was not tested here.
- In this patient, the effect of corneal collagen CXL on disease progression could not be established from a single postoperative Pentacam examination; serial pre- and postoperative topography is needed to judge treatment response in ectasia associated with systemic disease.
- Corneal topography may be considered in patients with POLR3-related disorders who have visual symptoms; systematic studies are needed before it can be recommended as routine screening.

## Data Availability

Original data generated and analyzed for this case report are included in this published article. The 2 *POLR3A* variants identified in this patient, NM_007055.4:c.2102G > T (p.(Gly701Val)) and NM_007055.4:c.2989C > T (p.(Pro997Ser)), were submitted separately to the NCBI ClinVar database as likely pathogenic, together with the relevant phenotypic and segregation evidence. The corresponding ClinVar accession numbers are SCV007670318 for c.2102G > T (p.(Gly701Val)) and SCV007670319 for c.2989C > T (p.(Pro997Ser)). The underlying whole-exome sequencing data were generated as part of clinical diagnostic testing and are not available to the authors for public deposition.

## Abbreviations

4H syndrome: hypomyelination, hypodontia, and hypogonadotropic hypogonadism
CXL: cross-linking; GnRH, gonadotropin-releasing hormone
HHG: hypogonadotropic hypogonadism
nc-RNA: noncoding RNA
PK: penetrating keratoplasty
Pol III: polymerase III; RNA, ribonucleic acid
SDS: standard deviation score
